# Effects of active musical engagement during physical exercise on anxiety, pain and motivation in patients with chronic pain

**DOI:** 10.3389/fpain.2022.944181

**Published:** 2022-11-22

**Authors:** Lydia Schneider, Ulrich Tiber Egle, Doris Klinger, Wolfgang Schulz, Arno Villringer, Thomas Hans Fritz

**Affiliations:** ^1^ Department of Neurology, Max-Planck-Institute for Human Cognitive and Brain Sciences, Leipzig, Germany; ^2^ Psychiatrische Klinik Sanatorium Kilchberg, Zürich, Switzerland; ^3^ Vitos Klinik für Psychosomatik, Weilmünster, Germany; ^4^ Department of Clinical Psychology, Psychotherapy and Diagnostics, Faculty of Psychology, Technische Universität Braunschweig, Braunschweig, Germany; ^5^ Institute for Psychoacoustics and Electronic Music (IPEM), University of Ghent, Ghent, Belgium

**Keywords:** chronic pain, physical activity, musical feedback, musical agency, anxiety, motivation

## Abstract

The experience of anxiety is central to the development of chronic pain. Music listening has been previously shown to exert analgesic effects. Here we tested if an active engagement in music making is more beneficial than music listening in terms of anxiety and pain levels during physical activity that is often avoided in patients with chronic pain. We applied a music feedback paradigm that combines music making and sports exercise, and which has been previously shown to enhance mood. We explored this method as an intervention to potentially reduce anxiety in a group of patients with chronic pain (*N *= 24, 20 female and 4 men; age range 34–64, *M = *51.67, *SD* = 6.84) and with various anxiety levels. All participants performed two conditions: one condition, *Jymmin*, where exercise equipment was modified with music feedback so that it could be played like musical instruments by groups of three. Second, a *conventional workout condition* where groups of three performed exercise on the same devices but where they listened to the same type of music passively. Participants' levels of anxiety, mood, pain and self-efficacy were assessed with standardized psychological questionnaires before the experiment and after each condition. Results demonstrate that exercise with musical feedback reduced anxiety values in patients with chronic pain significantly as compared to conventional workout with passive music listening. There were no significant overall changes in pain, but patients with greater anxiety levels compared to those with moderate anxiety levels were observed to potentially benefit more from the music feedback intervention in terms of alleviation of pain. Furthermore, it was observed that patients during *Jymmin* more strongly perceived motivation through others. The observed diminishing effects of *Jymmin* on anxiety have a high clinical relevance, and in a longer term the therapeutic application could help to break the Anxiety Loop of Pain, reducing chronic pain. The intervention method, however, also has immediate benefits to chronic pain rehabilitation, increasing the motivation to work out, and facilitating social bonding.

## Introduction

Pain is a highly subjective experience that is universally perceived by human beings and informs us about potential health problems. However, pain is sometimes persistent and in duration exceeds the normal healing process (1), occasionally leading to chronic pain. This has become a major health issue throughout the world. Around 20% of the adult population of developed countries at some point in their life suffer from chronic pain and its profound effects on their quality of life (2). Furthermore, chronic pain is associated with high emotional distress, fatigue, physical disability (e.g., limited in their general activity), cognitive and psychological impairments (e.g., depression, anxiety) and social isolation (3). These factors lead to a cumulative allostatic load (a composite index of indicators of strain on organs and tissues) in chronic pain patients (4), which has an accelerating effect on the aging process with respect to cognitive and physiological functionality (5).

Chronic pain may have different mechanism of etiopathogenesis, which makes a thoroughly diagnostic process important, if necessary in an interdisciplinary context of medicine. It is for example necessary to differentiate between nociceptive–neuropathic pain and stress-induced pain, because different treatments are considered differently effective depending on the type of pain. Stress-induced pain seems to be initiated without nociceptive lesions (6, 7).

### Avoidance behavior and anxiety hinder recovery

The interplay of pain, physical disability and psychological impairment (especially depression and anxiety) creates a vicious cycle of avoidance behavior in chronic pain patients. In its most simple form, the perception of pain symptoms (such as muscle tension, stiffness, etc.) leads to less physical activity (avoidance behavior) that over time leads to a reduction of physical fitness and capabilities, which in turn leads to the perception of more/greater pain symptoms (8). Therefore anxiety and fear of pain play a central role in the development of chronic pain, probably even more than the intensity of the pain sensation itself (9).

**Figure 1:**
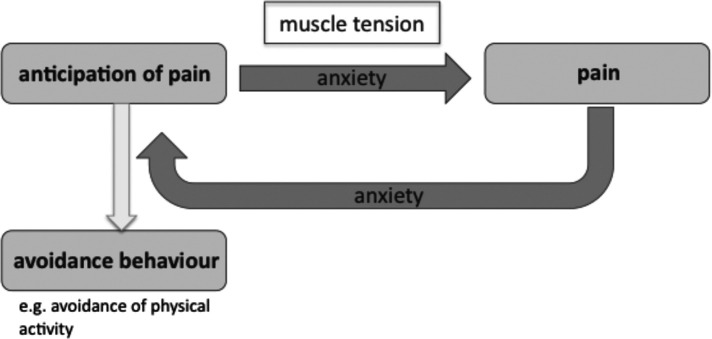
Anxiety loop of pain.

Furthermore, recent evidence shows that anxiety mediates the vicious cycle between an anticipation of pain and pain [Figure 1 (10)]. This study demonstrated that heightened anxiety in patients with chronic wounds is associated with the quantity of newly developed wounds, heavy exudation, and wound necrosis. This finding has been discussed in terms of a mechanism the authors called “nocebo hyperalgesia”, whereby anticipation of pain causes an emotional anxiety response that leads to a nocebo-like generation of pain.

### Benefits of physical activity in the treatment of chronic pain

A number of clinical studies have demonstrated the positive effects of regular physical activity in the treatment of nearly all types of chronic pain (3) and exercise therapy is recommended as a first-line treatment in the management of chronic non-specific low back pain by the European Guidelines (11). Additionally, recommencement of physical activity is important for patients with chronic pain in terms of promoting general health, well-being and quality of life (12). Evidence furthermore indicates that physical exercise activates central pathways associated with an opioid-mediated analgesia (13).

### Perceived barriers to physical activity

Chronic pain is associated with a psychomotor slowing and an overall “stiffening effect”, which adds to the patients' sensation that physical activity is highly exhausting and fatiguing (14). Furthermore, depression and anxiety, both highly prevalent in chronic pain patients, foster physical disability and limit general level of patient activity (15). Depression has been shown to be associated with a decreased level of physical exercise (16). Anxiety has been suggested to be instrumental for a number of negative effects on perceived pain: Lowering the pain threshold (17), increasing attention to the pain sensation (18), and creating the percept of tightness and constriction in the patient (19). This renders it rather unlikely for the patient to engage in exercise and enjoy physical activity. Moreover, the limiting effect of movement disability on general activity in chronic pain patients leads to a deterioration of mood and social functioning, which increases the risk to develop psychological comorbidities such as depression (3).

### Efficacy of musical interventions in the management of chronic pain

Recently, the use of music as a component of chronic pain intervention has gained greater importance. A number of clinical studies on chronic pain have shown that music listening can lead to reduced stress and pain levels (20–22). For example, pain-reducing effects associated with music listening have been shown in patients with fibromyalgia syndrome, where pain is perceived in the fibrous tissues of the body, such as muscles, tendons, and ligaments (20). The authors have argued that the observed analgesic effects of music in this study may partly be due to emotional effects related to the perception of pleasure and relaxation (20).

Another recent study showed a reduction in pain, anxiety and depression in chronic pain patients who underwent daily music listening sessions during hospitalization and at home (this intervention however also included describing their experiences at the end of each listening session to care staff (22);. Pain-reducing effects were reported by the participants to sustain for up to one month after the last music intervention session.

The precise mechanisms underlying the observed music effects on chronic pain are still unclear. Research of musical effects on pain (not chronic pain) suggest that music may decrease pain sensation *via* a release of endorphins and changes in the catecholamine levels; it may also be beneficial in this respect by diverting attention from the experience of pain (23, 24). This endorphin theory of musical analgesia has been further elaborated in a study where musical agency due to singing, clapping, instrument playing etc. was associated with a increase in pain tolerance, probably also in relation to enhanced endorphin release by high-energy musical activities (25). However, it is unclear how such musical effects may be called upon when addressing effects on chronic pain.

### Benefits of the interaction of music and sports in pain

Only a few studies have addressed the investigation of pain perception combining physical activity with music (26). In a repeated measure single-case series of patients with fibromyalgia an influence of music and walking speed on pain level was examined. While an average gait speed was observed to be higher with fast music and lower with slow music, an increase in walking speed was not associated with pain increase (26). In a recent study a significant increase in pain threshold as measured with the cold pressor task was demonstrated as an effect of musical agency during fitness machine workout (27). In this paradigm fitness equipment was modified so that participants could use the fitness equipment as musical instruments, jointly creating a musical performance as part of the fitness workout.

### Research questions and hypotheses—possible benefits of workout with musical agency in chronic pain management

In the present study we investigated the effects of a novel music-sports intervention, which allows participants to be musically expressive by operating on fitness machines. This intervention has been called *Jymmin*. In previous studies it has been shown that perceived exertion is decreased and motor efficiency and muscle relaxation increased when combining workout with musical agency in such a way compared to a control condition where music was listened to passively during workout (*conventional workout*). These observed physiological effects were discussed in terms of increased emotional motor control during the musical agency condition (28).

In a further study with a similar *Jymmin* paradigm, participants reported an enhanced mood after *Jymmin* compared to conventional workout with music listening (29). This may be especially relevant to an intervention with chronic pain patients, given that 63% of patients suffering from severe pain have previously reported the need to enhance their mood [results of the European survey of chronic pain patients (2)]. In addition, the observed mood enhancing effects due to fitness training with musical feedback also suggests that musical agency makes the training on workout machines more desirable (29). In this previous study also an investigation of anxiety was addressed, that in the investigated student population did not show an effect. Authors argued that this might be due to a ceiling effect given the relatively low anxiety of participants to begin with and that it would be interesting to repeat the experiment with a high anxiety cohort (29).

Based on the above-mentioned positive effects of a combination of exercise machine workout and musical agency*,* the present study aimed at investigating its possible benefits on chronic pain management. Pain management has been described as the “intention to modulate patients’ pain or their response to pain using multimodal approaches in a collaborative relationship with the patient” with a focus on self-efficacy and patients' participation (1).

Given the complexity of chronic pain and its underlying psychological mechanisms, recent research suggests to more strongly consider co-occurrences/co-morbidities in pain patients and especially to more systematically address a role of depression and anxiety in the treatment of chronic pain (30, 31). Accordingly, in the current study we addressed both an investigation of chronic pain levels and psychological parameters that have been directly implicated in the development of chronic pain. These parameters were investigated according to the following hypotheses - note that the *experimental* condition is named *Jymmin* condition and includes a workout on fitness machines with musical feedback, while the *control* condition is named *conventional workout* condition as it includes workout on fitness machines with passive music listening:

1. The difference of baseline anxiety score and anxiety score after *Jymmin* significantly differs from the difference of baseline anxiety score and anxiety score after *conventional workout*; anxiety decreases more strongly after *Jymmin*.
2. The difference of baseline mood score and mood score after *Jymmin* significantly differs from the difference of baseline mood score and mood score after *conventional workout;* mood increases more strongly after *Jymmin*.
3. The difference of baseline pain level and pain level after *Jymmin* significantly differs from the difference of baseline pain level and pain level after *conventional workout*; pain level decreases more strongly after *Jymmin*.
4. The difference of baseline locus of control score and locus of control score after *Jymmin* significantly differs from the difference of baseline locus of control score and locus of control score after *conventional workout*; the external locus of control decreases and the internal locus of control increases more strongly after *Jymmin*.
5. The difference of baseline generalized self-efficacy score and generalized self-efficacy score after *Jymmin* significantly differs from the difference of baseline generalized self-efficacy score and generalized self-efficacy score after *conventional workout*; the generalized self-efficacy increases more strongly after *Jymmin*.

In addition to these hypotheses, exploratory analyses were used to gain further insights into how different parameters of the intervention may relate to each other and how training-related effects were perceived by participants, immediately and one day after the intervention (self-constructed items and follow-up questions).

## Methods

### Participants

**Figure 2:**
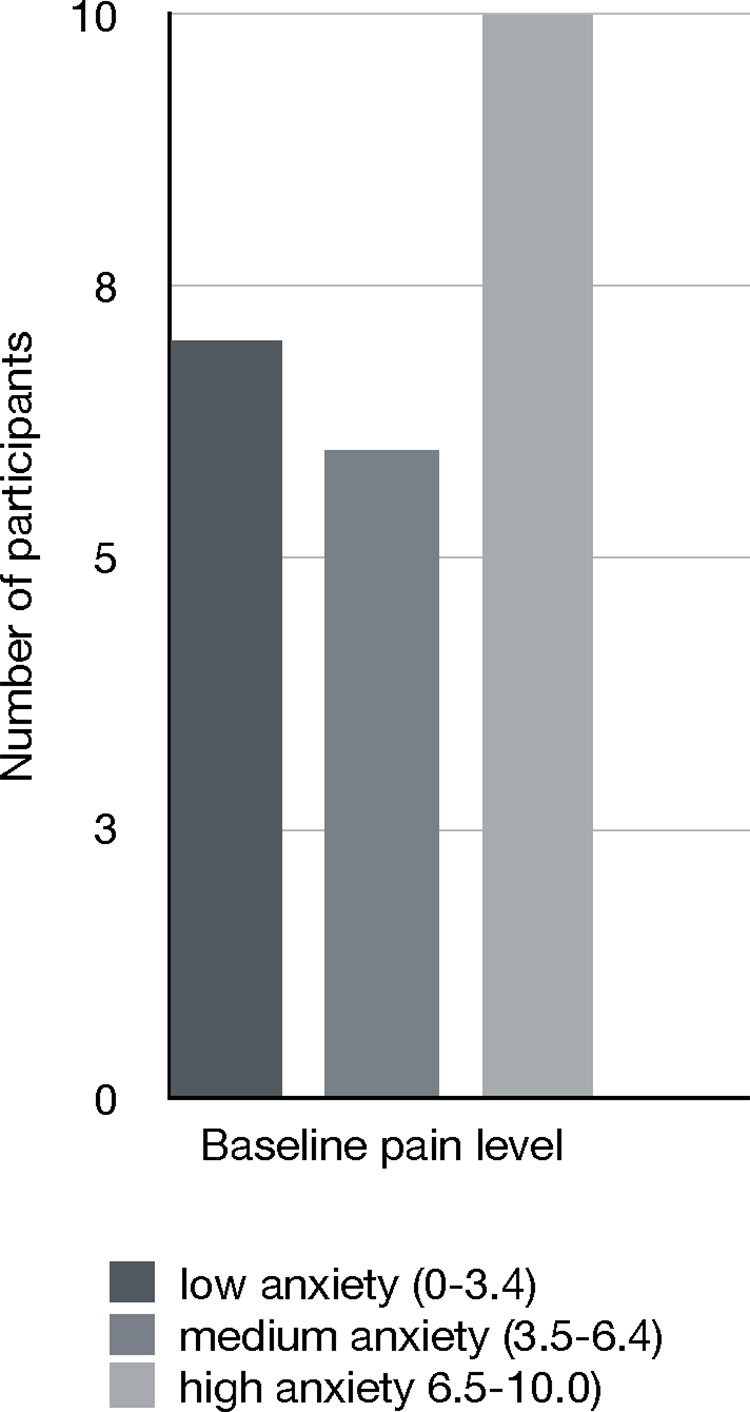
The figure depicts patients’ pain levels as measured on a visual analog scale (1–100 mm) before the intervention.

Twenty-four participants (20 female and 4 men; age range 34–64, *M = *51.67, *SD* = 6.84) took part in the experiment. None of the participants were professional body builders, musicians, or athletes. Clinical data showed that all participants were suffering from chronic pain, which has been defined as pain that persists or recurs for more than three months, with no clear physical causes (e.g., stress-induced pain conditions). Co-morbid conditions were present in the majority of patients as assessed with the *Brief Patient Health Questionnaire* (32); see section of experimental procedure): 34.8% of participants showed a depressive or major depressive syndrome, 4.4% of participants classified for a panic syndrome, and 21.7% showed both a depressive/ major depressive and panic syndrome. Patients' scheduled discharge date of the clinic varied between one to four weeks (*M *= 2.7, *SD *= 1.3). On the day of the experiment, patients' pain levels ranged from 0.3 to 9.4 (*M *= 5.14, *SD* = 3.04) as indicated on a Visual Analog Scale of 0–100 mm. 30.4% (*n* = 7) of participants suffered from mild pain, 26.1% (*n* = 6) from moderate pain, and 43.5% (*n* = 10) from severe pain (Figure 2). Inclusion criteria were any type of chronic pain and the ability to perform a physical exercise intervention. An exclusion criterium was accordingly a disability to perform physical exercise. Participants were referred by their treating therapist/doctor at the rehabilitation centre.

The study adhered to the guidelines of the Declaration of Helsinki and was approved by the ethics committee of the University of Leipzig, Germany. In addition, informed consent was obtained from all participants from the clinic.

### Experimental design

The experiment included two conditions: In one condition the participants worked out on fitness machines while passively listening to music (*conventional workout condition*); in a second condition they worked out on fitness machines while listening to a musical feedback of their own movements (*Jymmin condition*). All participants performed both conditions of the experiment, the *Jymmin condition* and the *conventional workout condition*. The physical workout was conducted with three different fitness machines, a tower (lat pulldown), a stomach trainer, and a stepper. All three machines are standard fitness machines that are commercially available and they allow for guided movements.

In the *Jymmin* condition the movements of participants on the fitness machines were mapped to a music composition software (Ableton Live 8) so that the deflection of the fitness machines was translated into musical parameters of an acoustic feedback signal [for a detailed description see (29, 28)]. Each fitness machine produced a different musical soundscape, and the combined musical feedback of all three fitness machines created sounds at a constant tempo of 130 bpm (beats per minute) and could interactively be combined into a holistic musical piece, which allowed for group performances of three participants in one group. The musical interaction was predefined in terms of sounds to be modulated, the musical parameters to be modulated, and the metric of the music. The music style used in the experiment was rather minimalistic electronic music. In the experiment one interactive musical composition was used, chosen by the experimenters. This consisted of musical elements that could be varied in a contained fashion determined by the composer so that participants had the experience that the more they exerted themselves with a certain movement on their respective fitness equipment, the greater the arousal of the musical element they controlled with sound, creating for each participant the experience that they could control their part of the interactive music composition expressively. The musical performances of all patient groups in the *Jymmin* condition were recorded and played back during the *conventional workout* condition (with exception of the first group who listened to a recording of their own *Jymmin* condition) to ensure a comparable exposure to the same musical piece during both experimental conditions. Furthermore, we controlled for the sequence in which both conditions were performed, so that half of the patients first performed *Jymmin*, and the other half first performed *conventional workout* (cross-over design).

### Experimental procedure

Patients suffering from chronic pain were recruited from a psychosomatic clinic and centre for stress-related diseases and pain disorders in Germany. Participants were randomly assigned to different time slots, such that eight groups (each consisted of three participants) were formed and tested on two consecutive days. Before participants started with the workout conditions, they were asked to fill out general information items on gender, age, and standardized questionnaires to assess a baseline of their physical and mental state before the experiment. The following questionnaires were given to the participants in the same order as presented here: Multidimensional Mood Questionnaire (MDMQ) (33), State-Trait Anxiety Inventory [STAI; the current study assessed only the state anxiety (34)], Pain Visual Analogue Scale (100 mm VAS), Rotter Internal-External Locus of Control Scale (I-E) (35), and the Generalized Self-Efficacy Scale (GSE) (36).

After the baseline assessment of the patients' physical and mental state, patients entered the training room and were asked to choose their preferred fitness machine (participants performed both conditions on the same fitness machine). A short explanation on how to use the fitness machines correctly in terms of physiologically healthy movements was given by the experimenter, followed by the task instruction: “Use the fitness machines now in a way in which you are physically comfortable.” Each of the conditions was performed for 10 min; during which all participants could hear the sound through a speaker system. After each condition patients took a rest and were then asked to fill-out the *MDMQ, STAI, VAS, I-E* and *GSE* questionnaires a second and third time. In addition the following self-constructed items relating to the training context were assessed after each condition.

#### Training related self-constructed items

Self-constructed items assessing subjective ratings of both training contexts were used to allow for additional explorative analyses*.* Training related self-constructed items assessed the perceived pain, impairment by pain and anxiety of pain during workout with the following nine questions: “How much did you perceive pain during workout?”, “how much did you suffer from pain during workout?”, “how much were you afraid that your pain would get stronger during workout?”, “how much did you feel impaired by pain during workout?”. Furthermore, it was assessed how well patients felt during workout in regard to their workout group and the music: “How comfortable did you feel in the group during workout?” and “how much did you like the music during workout?”. In addition, patients were asked about their motivation to workout: “How motivated did you feel?”, “how much did you feel motivated by your fellow training partners?” and “how much do you think this type of training could help you to exercise despite the experience of pain?”. Answers were given on a Visual Analogue Scale ranging from 1 (“not at all”) to 100 mm (“very strongly”). At the end of the experiment patients filled out the *Brief Patient Health Questionnaire (Brief-PHQ* (32); to assess possible co-morbidities that are known to be common in chronic pain.

#### Follow-up questionnaire

For further insights about how the intervention was perceived by patients with chronic pain, we used a follow-up questionnaire, which allowed for additional explorative analyses. In this follow-up questionnaire one day after the experiment participants were asked to answer intervention-specific questions on physical and psychological experiences during the remainder of the previous day: “To which extent did you experience positive physical effects during the remainder of the day after the workout intervention?”, “to which extent did you experience positive mood effects during the remainder of the day after the workout intervention?”, “to which extent did you experience an improvement in bodily relaxation during the remainder of the day after the workout intervention?”, “to which extent did you experience an improvement in mental relaxation during the remainder of the day after the workout intervention?” and “how much were you impressed by yourself actually performing a 20 min workout?”. In addition, anticipation and motivation to perform workout with musical agency were assessed: “How would you describe your anticipation of *Jymmin*?”, “how much do you think it could help you to perform *Jymmin* regularly?” and “how much would you like to use *Jymmin* at home?”. Answers were given on a Visual Analogue Scale ranging from 1 (“not at all”) to 100 mm (“very strongly”).

### Data analysis

The behavioral data were analyzed with non-parametric tests using SPSS 22 (IBM). Guidelines for missing responses on standardized questionnaires were applied as indicated in the corresponding questionnaire manuals. If participants had too many missing responses in the questionnaires, more than the corresponding guidelines allowed for, they were indicated in SPSS and excluded from the corresponding analysis. In order to obtain mean scores for the subscales of the *MDMQ* and locus of control questionnaire, responses for each subscale were averaged. In total, data from the *pain VAS* were analyzed for 22 participants, data from the *STAI* for 17 participants, data from the *MDMQ* for 16 participants, data from the *I-E scale* for 23 participants, and data from the *GSE* for 21 participants. For hypotheses testing in the current study, Wilcoxon Signed Rank Tests were applied. A Bonferroni-correction was applied to account for multiple comparisons, resulting in a significance level of *p* = 0.01 (.05 divided by the 5 hypotheses tested in this study).

## Results

### Results of standardized psychological questionnaires

**Figure 3:**
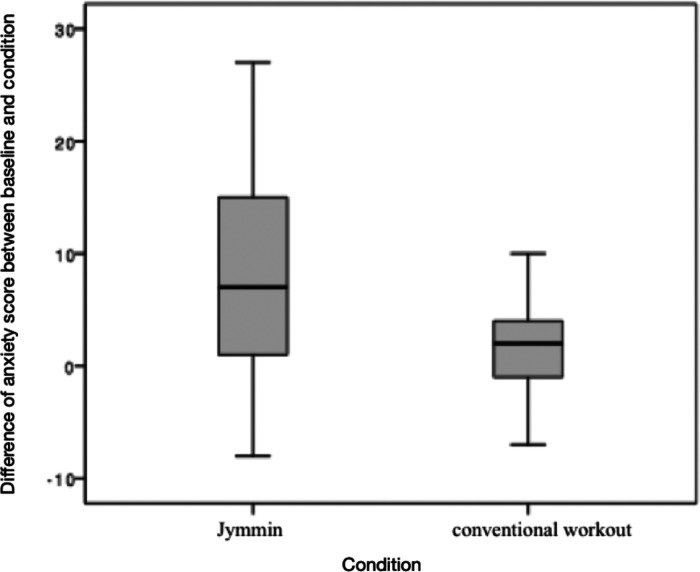
The figure depicts differences of baseline anxiety scores and the anxiety scores after *Jymmin* or *conventional workout* condition as measured with the *STAI*. A Wilcoxon Signed Rank Test revealed a significant difference between medians of the difference between baseline and the *Jymmin* condition, and the difference between baseline and the *conventional workout* condition. Anxiety total scores declined stronger after the *Jymmin* condition as compared to the *conventional workout* condition.

A Wilcoxon Signed Ranks Test for dependent samples was applied to test hypothesis 1 that anxiety decreases more strongly after *Jymmin*. Results showed that the *difference of baseline* anxiety score and anxiety score after *Jymmin* (*Mdn *= 7.00) differed significantly from the *difference of baseline* anxiety score and anxiety score after *conventional workout* (*Mdn* = 2.00). Anxiety decreased significantly stronger after the *Jymmin* condition, *Z* = −2.520, *p* = 0.006 (one-tailed), *r *= 0.43 (see Figure 3). Note that no sequence effect was found for differences in anxiety scores using the Mann–Whitney *U* Test.

An additional exploratory analysis was performed to gain further insight about how anxiety may have influenced other relevant aspects of physical engagement. Spearman's correlations were carried out to investigate if anticipation of pain is related to perceived pain as discussed in previous studies (10). Spearman's correlation matrices showed that being anxious about experiencing pain during workout correlated positively with the patients' pain level during both exercising in the *Jymmin* condition (*N* = 22, *r_s_* = 0.706, *p* < 0.001) and *conventional workout* condition (*N* = 23, *r_s_* = 0.500, *p* = 0.015). Being anxious about experiencing pain during workout also correlated both with the degree to which patients felt impaired by their pain during the workout on fitness machines during the *Jymmin* condition (*N* = 23, *r_s_* = 0.840, *p* < 0.001) and the *conventional workout* condition (*N* = 23, *r_s_* = 0.847, *p* < 0.001). Interestingly, being anxious about experiencing pain during workout correlated negatively with feeling comfortable in the group during the *conventional workout* condition (*N* = 23, *r_s_* = −0.481, *p* = 0.020), but not during the *Jymmin* condition (*N* = 23, *r_s_* = 0.061, *p* = 0.781).

A Wilcoxon Signed Ranks Test for dependent samples was applied to test hypothesis 2 that mood increased more strongly after *Jymmin*. Results revealed that the difference of baseline mood score and mood score after *Jymmin* (*Mdn *= −3.00) did not differ significantly (after Bonferroni-correction) from the difference of baseline mood score and mood score after *conventional workout* (*Mdn *= 1.00)*, Z* = −2.171, *p* = 0.015 (one-tailed), *r* = 0.38, as measured with the *MDMQ*. Again, no sequence effect was found.

Furthermore, to test the hypothesis 3 that pain levels decrease more strongly after *Jymmin* a Wilcoxon Signed Ranks Test for dependent samples was applied. Results revealed that the difference of baseline pain level and pain level after *Jymmin* (*Mdn *= 0.30) did not significantly differ from the difference of baseline pain level and pain level after *conventional workout* (*Mdn *= 0.10), *Z* = −1.429, *p* = 0.074 (one-tailed) as measured with the *pain VAS*. No sequence effect was found for differences in pain level using the Mann–Whitney *U* Test.

**Figure 4:**
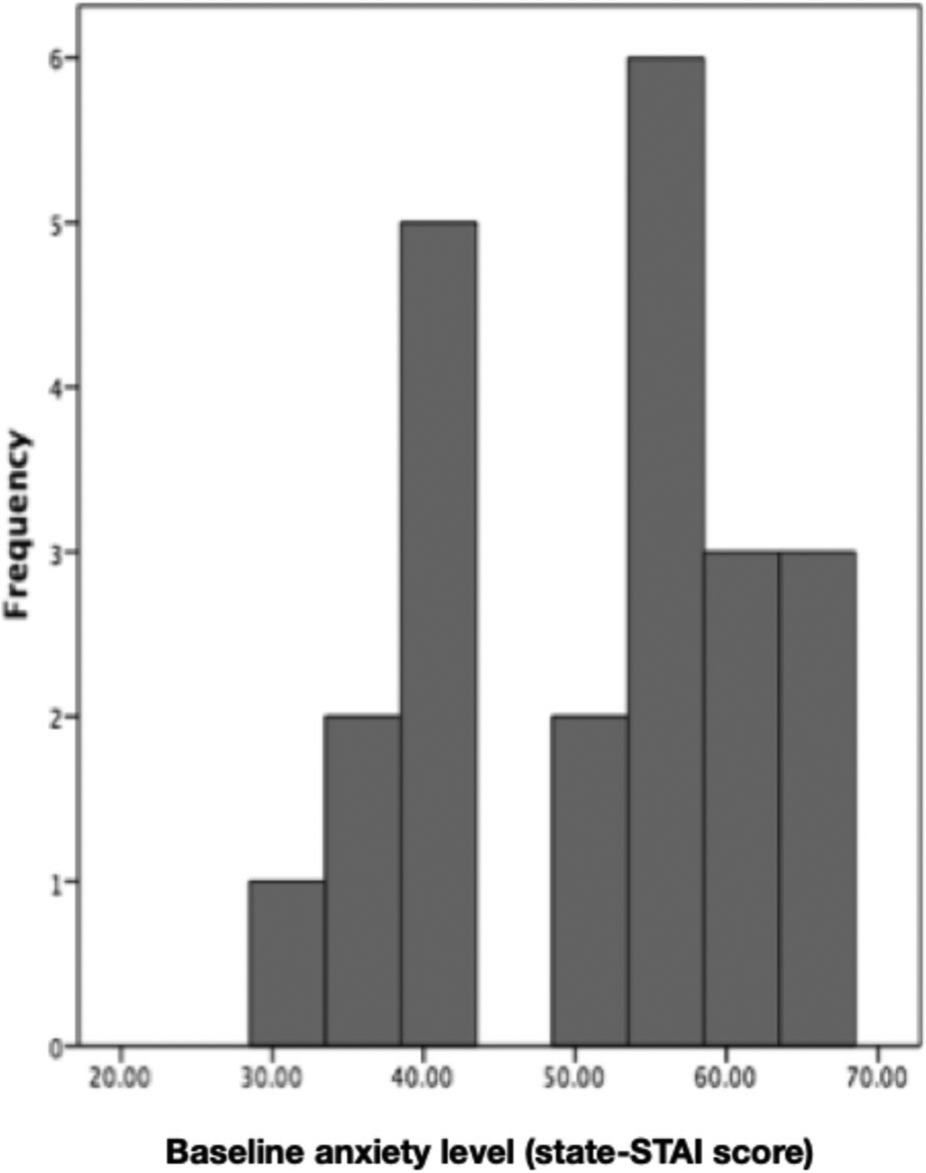
The figure depicts baseline anxiety levels of participants.

Interestingly, the level of anxiety before the patients started the intervention could play a role on the interventional effects on pain perception. We performed a subsequent analysis where we analyzed two subgroups of patients separately: patients with medium anxiety (STAI ≤ 45; *n* = 8) and patients with high anxiety levels (STAI > 45, *n* = 14; Figure 4). Descriptive statistics seem to suggest that those patients who had high anxiety levels benefitted from both conditions in terms of pain reduction, and to a stronger degree from the *Jymmin* condition. However, those patients who reported medium levels of anxiety seem to display a trend of increases in pain perception after both conditions (Table 1). Note however, that differences are not significant.

**Table 1:** Descriptive statistics on levels of anxiety and changes in pain perception.

Anxiety level	*n*	Pain level at baseline	Pain level after Jymmin	Pain level after conventional workout
Medium anxiety	8	Mdn = 4.25	Mdn = 4.80	Mdn = 5.85
		Var = 9.37	Var = 7.43	Var = 4.73
High anxiety	14	Mdn = 6.85	Mdn = 4.55	Mdn = 5.70
		Var = 7.59	Var = 7.98	Var = 5.01

Medians and variances of pain level for both experimental conditions are displayed for patients with medium anxiety levels at baseline and for patients with high anxiety at baseline.

In addition, a Wilcoxon Signed Ranks Test was applied to test hypothesis 4: External locus of control decreases and the internal locus of control increases more strongly after *Jymmin*. Results showed that the difference of baseline external locus of control score and external locus of control score after *Jymmin* (*Mdn *= 0.00) did not differ significantly from the difference of baseline external locus of control score and external locus of control score after *conventional workout* (*Mdn *= 0.00), *Z* = −0.182, *p* = 0.428 (one-tailed), nor did sequence have an effect on the outcome. The difference of baseline internal locus of control score and internal locus of control score after *Jymmin* (*Mdn* = 0.00) did not significantly differ from the difference of baseline internal locus of control score and internal locus of control score after *conventional workout* (*Mdn *= 0.00), *Z* = 0.000, *p* = 0.500 (one-tailed). Again, sequence did not affect the results.

Similar results were found for the *generalized self-efficacy scale*. A Wilcoxon Signed Ranks test was applied to test hypothesis 5 that self-efficacy increases more strongly after *Jymmin*. The difference of baseline self-efficacy score and self-efficacy score after *Jymmin* (*Mdn* = −1.00) did not differ significantly from the difference of baseline self-efficacy score and self-efficacy score after *conventional workout* (*Mdn *= −2.00), *Z* = −0.856, *p* = 0.196 (one-tailed), nor did sequence has an effect on the results.

### Additional explorative analyses-results of training related self-constructed items and follow-up questionnaire

**Figure 5:**
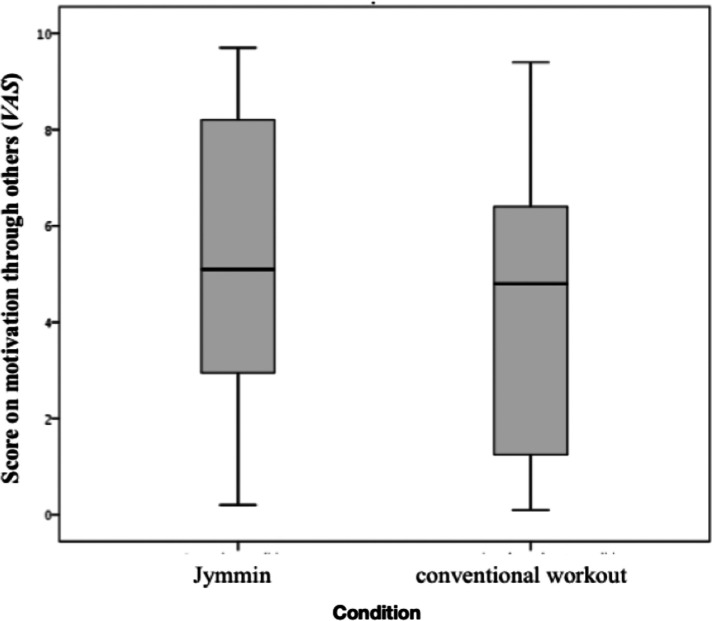
The figure depicts scores on the motivation through others during the *Jymmin* or *conventional workout* condition as measured on a *VAS*. A Wilcoxon Signed Rank Test revealed significant difference between medians of the *Jymmin* and *conventional workout* condition. The motivation through other training partners was significantly higher during *Jymmin* as compared to *conventional workout*.

Explorative analyses on training related items and follow-up questionnaires were performed using Wilcoxon Signed Ranks Tests. Note that in explorative analyses two-tailed *p*-values were calculated and no Bonferroni correction was applied. Results showed significant effects of musical agency on motivation mediated through the group experience. In the *Jymmin* condition (*Mdn* = 5.10) patients (*N = 21*) were significantly more motivated through other group members than during the *conventional workout* condition (*Mdn* = 4.80), *Z* = −2.660, *p* = 0.008 (two-tailed), *r *= 0.41 (see Figure 5). Self-motivation did not differ significantly between *Jymmin* (*Mdn* = 6.50) and *conventional workout* (*Mdn* = 6.70). Furthermore, the patients (*N* = 23) liked the music during the *Jymmin* condition (*Mdn *= 4.50) significantly more than during the *conventional workout* condition (*Mdn *= 2.90), *Z* = −2.766, *p* = 0.006 (two-tailed), *r *= 0.41.

**Figure 6:**
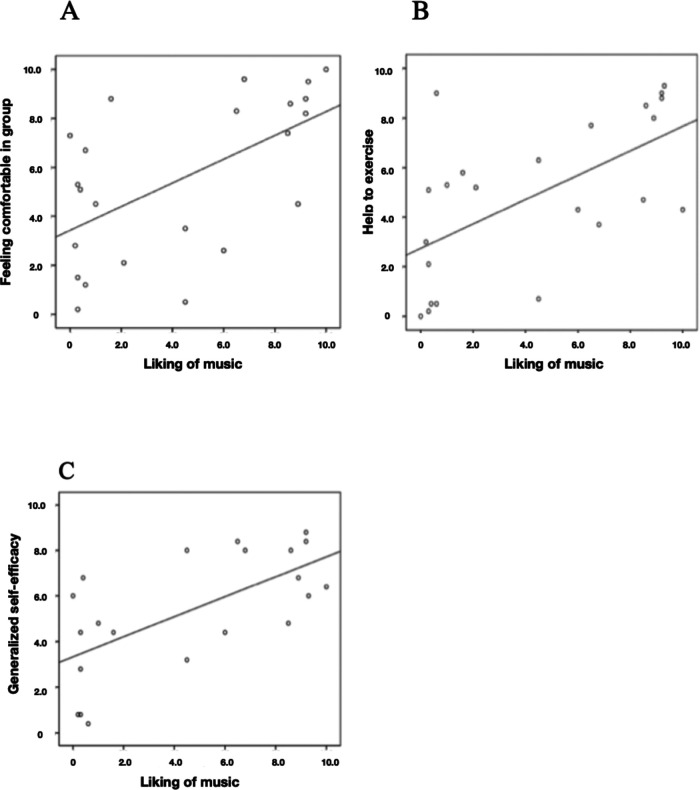
The figure depicts Spearman's correlations of (**A**) how liking the music during the *Jymmin* condition correlated with how comfortable they felt within the training group, (**B**) how much liking the music during *Jymmin* correlated withy how much they thought *Jymmin* would be an incentive to exercise despite having pain, and (**C**) how liking the music during *Jymmin* correlated with generalized self-efficacy scores after performing *Jymmin* as measured with the GSE.

In addition, liking the music in the *Jymmin* condition correlates significantly positive with feeling comfortable in the group (*N* = 23, *r_s_* = 0.594, *p* = 0.003) and how *Jymmin* was perceived as an incentive to do sports despite the feeling of pain (*N* = 23, *r_s_* = 0.629, *p* = 0.001). Moreover, liking the music during *Jymmin* correlates positively with generalized self-efficacy scores after the *Jymmin* condition (*N* = 21, *r_s _*= 0.630, *p* = 0.002; Figure 6), but liking the music during *conventional workout* does not show such a correlation (*N* = 23, *r_s _*= 0.218, *p* = 0.317).

Results of the follow-up questionnaire were assessed with visual analog scales (1–100 mm). Note that because participants performed both conditions as part of the experimental cross-over design, no differentiation of the following assessments was made between conditions. Furthermore, responses of 50 or higher were defined as medium to high effects. For each item of the questionnaire the percentage of participants was calculated who responded in the range above 50. 100% of the participants was defined as the total number of patients who took part in the experiment (*N* = 24). Patients who did not fill out the items in the questionnaire were considered in the analysis as if they had responded lower than 50. Results of the follow-up questionnaire showed that more than one third of the patients (37.5%) were experiencing medium to high positive physical effects during the remainder of the day after the workout intervention. Furthermore, 41.6% of all patients reported a medium to high positive effect on their mood during the rest of the day. The experience of an enhanced mood lasted for 0.5–7 h after the workout (*N *= 14, *M* = 3.39, *SD* = 2.19). In addition, bodily and mental relaxation was reported by about one third of the patients (33.4%).

Results on questions regarding the *Jymmin* intervention revealed that 66.7% of the patients had a positive attitude towards the *Jymmin* intervention (scale of 50 or higher). Furthermore, 45.6% of the patients believed that *Jymmin* could help them (scale of 50 or higher) to exercise regularly. In addition, 37.5% of the patients would like (scale of 50 or higher) to do *Jymmin* at home. Overall, over half of the patients (54.2%) were positively surprised by themselves to have sustained a fitness training for a total duration of 20 min.

## Discussion

**Figure 7:**
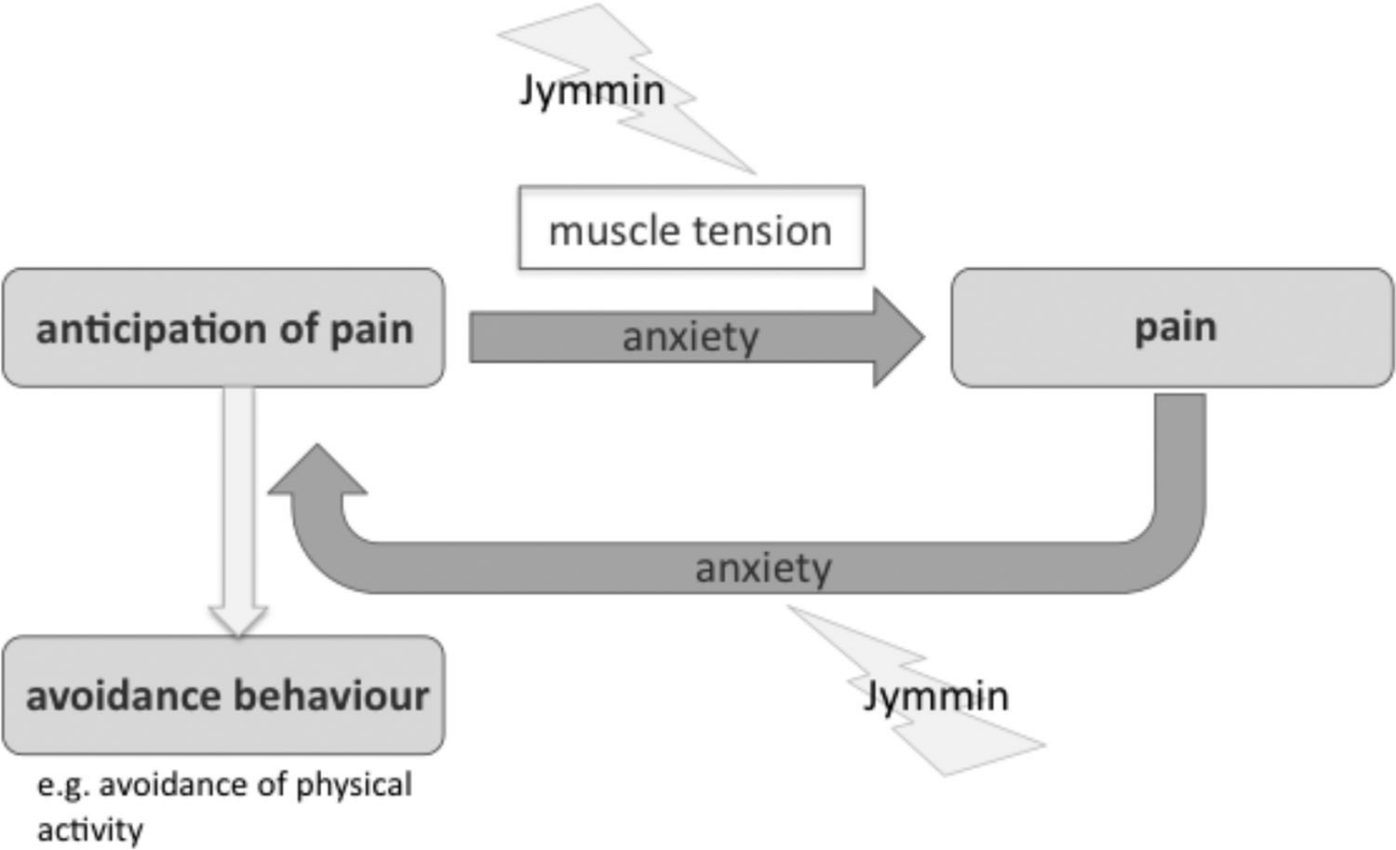
Influence of *Jymmin* on the Anxiety Loop of Pain. Disruptive effect of *Jymmin* on the depicted loop are indicated by the lightning symbol.

Results show that patients who worked out with musical feedback during the *Jymmin* condition had significantly reduced anxiety levels compared to when they exercised in the *conventional workout* condition, in which they passively listened to music while working out (status quo in fitness training). Physical activity is known to be highly beneficial in the treatment of chronic pain, for example increasing the patients' overall quality of life (12). However, patients' anticipation of pain tends to lead directly to avoidance behavior so that they do not engage in physical activity. Alternatively, when they do engage in physical activity, their anticipation of pain tends to lead to a state of anxiety and tension, which renders exercise less effective, more exhausting and more painful. As illustrated by the Anxiety Loop of Pain (Figure 1), (state) anxiety plays a crucial role in the avoidance of physical activity in chronic pain patients, amplifying the feedback between pain and the anticipation of pain. The current results therefore are highly relevant to our understanding of how this vicious cycle (Anxiety Loop of Pain) may be broken (Figure 7).

Two mechanisms may account for the observed effect of musical agency during exercise on anxiety. First, previous evidence suggested that workout with musical feedback/agency led to a higher metabolic muscle efficiency, which is associated with greater muscle relaxation [less activation of the antagonist muscle (28);]. Muscle relaxation corresponds to a decrease in muscle tension, which has been described to be one of the most prominent physiological signals directly related to the subjective percept of anxiety [other less salient cues comprise parameters of the stress reaction such as blood pressure (37);]. Given that muscular tension in chronic pain patients can be seen as a psycho-physiological correlate of learned fear (38) such a decreasing effect on muscle tension by doing *Jymmin* is likely to also have had an influence on their subjective percept of anxiety. In the current study we investigated a one-time intervention, we expect that the observed positive effects on anxiety would help to positively condition the movement experience with multiple use and could therefore be a useful approach to help chronic pain patients deal with avoidance behavior. However, this still remains to be tested in further studies.

Second, an alternative explanation for the observation that anxiety is reduced after *Jymmin* may be that an increased cognitive demand due to musical agency and a guided attention to the whole movement and involved body parts *via* the immediate music-feedback may have positively altered the attentional state such that they have less capacity/attentional resources to monitor pain symptoms and focus on possible pain experiences.

The above finding demonstrates that in the management of chronic pain it should be helpful to consider the influence of anxiety. This is especially the case when trying to engage patients to join and enjoy physical activities. Given its emotionally engaging and largely positive effects in listeners music is thus a powerful tool to facilitate this process.

Results of the present study showed that pain levels of patients did not change from baseline in either condition. This is surprising, given that music listening has previously been observed to ameliorate perceived pain in chronic pain patients (20, 22), and we had hypothesized that musical agency would decrease pain levels during the sports intervention. This may relate to two aspects, first the music presented in previous studies was either calming music aimed at inducing relaxation, or music that participants favored. These participants thus probably perceived this music as relatively pleasant, whereas in the present study the perceived valence of the utilized music (which was pre-selected and aimed at energizing participants for the sports workout) varied strongly between participants.

Second, in previous studies reporting positive effects of music listening the chronic pain patients only listened to the music and they were not required to exercise on fitness machines as it was the case in the present study. In each of the two sports interventions applied (and generally in every sports intervention), patients both anticipate and perceive discomfort and physical pain. Therefore it is plausible that with respect to pain the effects of making sports counteract those of musical analgesia. Indeed it seems somewhat surprising that in the present study pain levels did not increase in either of the sports conditions (that were both associated with music-actively making music or passively listening to music). Given that the vicious cycle between anticipation of pain and pain is mediated by anxiety (illustrated in Figure 7) the observed decrease in anxiety in both experimental conditions (but stronger during *Jymmin*) may have contributed to such a lack of effect of exercise on pain levels. Note that this is in accord with descriptive values that showed a non-significant trending in the predicted direction rather indicating a stronger decrease in pain after *Jymmin.* In addition, a subsequent analysis showed that patients with high anxiety levels at baseline seem to experience a greater benefit from the Jymmin condition in terms of pain reduction as compared to the conventional workout condition. Furthermore, greater variances in pain levels after Jymmin were observed. It may be interesting to investigate in further studies with a greater number of participants, which characteristics of patients with chronic pain need to be considered in order to differentiate for which patients the described music intervention is effective.

Furthermore, the present study aimed at investigating possible effects of musical agency during exercise on locus of control and generalized self-efficacy in chronic pain patients. No such effects on locus of control and self-efficacy were observed. However, a significant correlation was observed that relates to generalized self-efficacy: Liking the music during *Jymmin* correlates positively with generalized self-efficacy scores after the *Jymmin* condition, whereas liking the music during *conventional workout* does not show such a correlation. In other words, patients who enjoyed the outcome of their musical agency perceived a transfer to generalized self-efficacy. Alternatively, it could be the other way around, such that those participants who had greater self-efficacy scores were also prone to more strongly engage in musical agency and as a result could enjoy the outcome of their effort (literally). It is plausible that in the *conventional workout* condition therefore the perceived aesthetic quality of the music had no relation to self-efficacy.

The current data show that when patients were musically expressive together in a group, this enhanced their motivation to exercise more strongly than working out within the same group while listening to music passively. The higher motivation through others is probably at least partly due to contagious processes on the motor and emotional level. These contagious processes are probably amplified by the circumstance that participants are committing themselves through their exertion to a common aesthetic goal. Given that humans are social animals, such a goal will be best achieved as a joint endeavor where participants encourage each other with the means available, and also where participants more strongly feel a social obligation to participate than during the conventional workout condition. Musical expression is known to be a strong motivator to engage groups of people in activities and is present in most if not all important social rituals and occasions. Accordingly participants probably used the musical expression available to them as a communicational tool to motivate other training partners for example by performing with more enthusiasm when others seemed to wear out.

### Limitations of the current study

A limitation of the current study is the relatively small number of patients that could be included in the data analyses due to a substantial number of missing responses in the standardized questionnaires. Chronic pain patients are known to suffer from cognitive impairments such as concentration and attention deficits. Therefore, computer-based questionnaires in which missing responses are immediately indicated to the participant might be a good option to address this issue in future research.

The current investigation did not include a condition where patients only performed exercise in the absence of music presentation. It would be interesting to compare pain levels after either of the two current exercise-and-music conditions (where music is either made actively or passively listened to) with such a control condition, where patients would probably perceive increased pain levels due to exercise (which they regularly report during sports exercise). However, note that including a control condition aimed at inducing pain in chronic pain patients would ethically have to be carefully considered.

Locus of control and generalized self-efficacy that have been investigated in the current study are rather stable over time and probably hardly changed by a one-time intervention. In future research it might be preferable to ask intervention specific questions on self-control and self-efficacy such as control over pain during and after the experimental intervention.

### Implications and future research

The present study shows how being musically expressive during exercise machine workout reduces anxiety levels in chronic pain patients. This finding is highly relevant to the management of chronic pain, because anxiety is a key factor in hindering chronic pain patients to engage in physical activities, mediating the vicious Anxiety Loop of Pain (Figure 7).

Two things are often perceived to cause anxiety in a chronic pain patient, (1) Engaging in physical activity, (2) Engaging in social interaction. While physical activity is usually perceived to amplify pain, social interaction is rather perceived as a challenge and additional burden as soon as pain arises. That both aspects may be combined to create a positive experience, getting less anxious while engaging both in a workout and a social activity, can serve as an example to the chronic pain patient that intense physical activity can be enjoyed socially. It is important to note that this activity can even be enjoyed without full pain relief, which in many chronic pain patients unfortunately is beyond reach. Thus the combination of workout and musical agency seems promising for chronic pain management, reducing anxiety and promoting physical activity.

The social aspect of the intervention presented here is highly important as it provides experiences of being motivated by others as well as feeling comfortable within a social group. Chronic pain patients often suffer from social isolation as a result of their decreased general activity level. In addition, they are afraid of being somewhere else than at home when strong pain arises. In a rehabilitation context, where group therapeutic interventions are regularly applied, a positive experience with such an “anticipation-of-pain” evoking activity could result in positive transfer effects to other group interventions.

Future research should address long-term effects of the music-sports intervention investigated here. It would be of great interest to examine how repeatedly breaking the vicious Anxiety Loop of Pain with *Jymmin* could with regular training over time systematically decrease chronic pain. This would then further increase the motivation of patients to perform physical workout, facilitating physical rehabilitation. In addition, long-term effects on mood should be assessed, as well as more general concepts of patients’ well-being and quality of life. This could be complemented assessing physiological data such as heart rate, blood pressure, heart rate variability, hormone levels etc., which would help to better understand mechanisms of how the musical feedback intervention relates to pain perception during physical exercise. It would also be relevant for future studies to analyze the impact of the current intervention for a prolonged time to evaluate its effect with respect to different stages of the development of chronic pain.

## Data Availability

The raw data supporting the conclusions of this article will be made available by the authors, without undue reservation.
